# Corpus-based analysis of shifts in China’s diplomatic stance across seven decades (1949–2018)

**DOI:** 10.3389/fpsyg.2022.1021410

**Published:** 2022-11-17

**Authors:** Xujun Tian, Xiaoqian Li

**Affiliations:** ^1^School of Foreign Languages, Shanghai Lixin University of Accounting and Finance, Shanghai, China; ^2^Institute of Corpus Studies and Applications, Shanghai International Studies University, Shanghai, China

**Keywords:** China’s diplomatic stance, shifts, Chinese diplomatic discourse, critical discourse analysis, corpus

## Abstract

This study investigates the changes in China’s diplomacy in the past seven decades based on the diachronic corpus of Chinese diplomatic discourse from 1949 to 2018 using corpus based critical discourse analysis theoretical insights. The study examines the targeted keywords used in the seven periods of Chinese diplomatic discourse and their significant collocations. The results show that China has worked with the United States, Japan, the United Kingdom, and some Asian, Latin American, and African countries to safeguard world peace while opposing war, establish good relations with these countries, and promote mutual development through cooperation. In addition, China’s diplomacy has experienced politics-oriented and economy-oriented eras and is now in an era of global sustainable development. Therefore, Chinese diplomatic discourse is the linguistic representation of China’s diplomacy, fundamentally determined by its national interests and changing social and historical situations domestically and internationally.

## Introduction

Diplomacy is “the conduct of relations between sovereign states through the medium of officials based at home or abroad, the latter being either members of their state’s diplomatic service or temporary diplomats” (Berridge and Lloyd, 2012, p. 97). The subject of diplomacy of an independent sovereign state is neither an individual nor an organization or a group, but the central government (Guan, 2000; Jin, 2007). China’s diplomacy is a direct source of the international community’s perceptions, evaluations, and understanding of China (Hu and Li, 2018). It showcases China’s foreign policies and initiatives, such as “Five Principles of Peaceful Co-existence” in the 1950s and the “Belt and Road Initiative” in the 2010s. It constitutes an essential part of China’s soft power, expands China’s influence home and abroad, and constructs China’s national image on the international stage. Diplomatic discourse is “the linguistic and discursive actions resorted [to] by the subject of diplomacy to express their international strategies and foreign policies in a certain historical period” (Hu and Li, 2018, p. 5). In addition, the diplomatic discourse has been regarded as “the most institutionalized political discourse of the highest level of formality” (Wu and Cao, 2011, p. 28). The Chinese diplomatic discourse is the linguistic representation of China’s diplomatic policies and domestic and international activities in which China has initiated or participated. It is also an important source for the international community to learn about China’s stances and attitudes toward domestic and international affairs.

China, being the largest developing country in the world, has been attentive to build and improve its international image through diplomacy. According to Wang (2003), China has tried, through its diplomatic discourse, to project its images as a peace-loving country, victim of foreign aggression, socialist country, bastion of revolution, anti-hegemonic force, developing country, major power, international cooperator, and autonomous actor to world in the past 40 years. It is suggested by scholars (Sun, 2020; Sun and Kong, 2020) China should strengthen its diplomatic capacity, improve the discursive system, and cooperate with different diplomatic subjects and fields globally with the international community to fight the pandemic to maintain its international image. To better disseminate its ideas to the international community, China must construct a diplomatic discourse system characterized by Chinese characteristics as well as a shared future for humankind (Sun, 2017, Lu, 2018, Ma and Ma, 2018). Studies (Xu, 2017; Liu, 2018; Afzaal et al., 2019, 2022a,b) show that differences in US and Chinese diplomatic press conferences reflect their different national interests, ideologies, and cultural traditions, and the increased use of competitive and antagonistic lexical items in China and India’s diplomatic discourse indicates the pluralism and instability between these two countries.

Previously, many studies focused on diplomatic or major events that happened in a short period, but a few studies have investigated China’s diplomacy as a whole and have examined China’s diplomacy within a comparatively longer timespan through Chinese diplomatic discourse. In this regard, this research, based on the diachronic corpus of Chinese diplomatic discourse over 1949–2018, divides China’s diplomacy into seven different periods to investigate its changes in China’s diplomacy over the past 70 years. The following questions are addressed:

1. What are the significant linguistic features of Chinese diplomatic discourse over these 70 years?
2. What are characteristics of China’s diplomacy of different periods? How has it changed in the past 70 years?
3. What factors have contributed to the changes in China’s diplomacy over different periods?

## Research design

Corpus based approaches are employed for the diachronic studies to analyze the discourse of over 25 to 30 years. The research period can be divided into several periods to examine the changes that occurred over time (Baker, 2004; Hu and Li, 2017). Primarily, this study divides China’s diplomacy into seven periods and then introduces the diachronic corpus used for the study and the primary data of the corresponding periods. As is generally accepted, discourse is the linguistic representation of social facts, and the study on discourse investigates the correlation between linguistic features and social realities and changes. This study also applies Fairclough’s three-dimensional model to analyze the interaction between linguistic patterns and social realities to examine the changes in China’s diplomatic discourses. With this model, this research adopts a corpus-based approach first to examine the linguistic features of Chinese diplomatic discourse as a whole to find out the top priorities of China’s diplomacy by scrutinizing the critical keywords of Chinese diplomatic discourse. Then it examines Chinese diplomatic discourse at different periods to analyze the characteristics of China’s diplomacy at different periods reflected through the linguistic patterns. Finally, it explores the social and historical factors that contributed to the changes in China’s diplomacy.

### Division of the study period

Jin (2007) suggests that the policies and strategies of a diplomatic entity are often adjusted at different times. Indeed, researchers can “divide a long historical period into several periods following the different eras or major historical events [that] happened” (Hu and Li, 2017, p. 70). For example, the third plenary session of the 11th Central Committee of the Communist Party of China in Beijing in 1978 was a landmark moment in the country’s history, as it signaled the start of the reform and opening up era (Yang, 2008; Wang, 2015). The 2008 Beijing Olympics were another important event in China’s history, as the world could see the extent to which the country had opened further to the outside world (Yang, 2006; Bing, 2008). Drawing insights from the abovementioned scholars and based on the development of China’s diplomacy over time, this study considers these two important events as nodes. It divides China’s diplomacy into three stages and seven periods, with a span of 10 years for each period.

The first stage (1949–1978) is the initial period of China’s diplomacy. During this stage, China made great efforts to establish diplomatic relations with other countries. The first stage is subdivided into three periods. In the first period (1949–1958), the first decade of the People’s Republic of China, China initiated its diplomacy as a newly established country focusing on the bilateral relations with international community. The second period (1959–1968) was characterized by the slow development of the Chinese economy, during which domestic development, especially economic development, slowed due to the influence of the Great Leap Forward Movement and the turning of China’s relations with Russia. Finally, the third period (1969–1978) was a special period in China’s history in which it experienced twists and turns in its politics, economy, and foreign relations.

In the second stage (1979–2008), a period that saw the development of China’s diplomacy, economic development was of prime significance. After implementing the reform and opening-up policy in 1978, China conducted more frequent and closer communication and exchanges with other countries to facilitate its economic development. Similar to the first stage, this period is subdivided into three periods. In the fourth period (1979–1988), the initial stage of China’s reform and opening up policy, the country started to open to the outside world, and its diplomacy played an increasingly important role in facilitating exchange and communication with the world. In the fifth period (1989–1998), the Chinese economy experienced rapid development as the opening-up and reform policy accelerated. Finally, in the sixth period (1999–2008), during which China opened up even further and conducted exchanges with most countries in various fields, the Chinese economy grew faster than ever.

During the third stage (2009–2018), the seventh period, China’s strength in all aspects increased constantly. It started to set agendas, formulate rules, and participate in international affairs as a significant global power. Its top diplomatic priority in this period was to build an advantageous and favorable international image.

### The corpus

The present study is based on the diachronic corpus of Chinese diplomatic discourse over 1949–2018, which spans the 70 years from the founding of the People’s Republic of China in 1949 to the present (2018). The texts in the corpus include the diplomatic speeches and remarks of Chinese presidents, premiers, and (vice) foreign ministers; official diplomatic documents; inter-state treaties; agreements, statements, and declarations; diplomatic negotiations; articles signed by major leaders and interviews with Chinese presidents and premiers; and diplomatic decrees and instructions. The corpus size is 2,142,156 tokens (words). Table 1 lists the details.

**Table 1:** Basic information of the corpus.

Period	Timespan	Types	Corpus size (tokens/words)
First	1949–1958	5,944	81,745
Second	1959–1968	5,005	62,577
Third	1969–1978	1,582	7,686
Fourth	1979–1988	4,318	67,855
Fifth	1989–1998	4,959	56,894
Sixth	1999–2008	9,489	293,044
Seventh	2009–2018	18,427	1,572,355
Total (tokens/words)			2,142,156

### Theoretical foundations

The study employs the theatrical underpinnings of critical discourse analysis (CDA) while taking Faircloughian three dimensional model (Fairclough, 1992). The model presents discourse as a social behavior and language as a tool for the verbal representation of social facts. CDA claims that social reality is represented through language and that discourse is an important means of social construction (Wodak and Meyer, 2016). Fairclough (1992) claims that a text should be analyzed from these three dimensions; at the “text” dimension, the linguistic analysis of the text is made; at the “discursive practice” dimension, which is the “interaction” in the “text-and-interaction” view of discourse, investigates the property of the text production and interpretation processes, including what types of discourse are selected and the way how they are combined; and at the “social practice” dimension, the issues of social analysis are examined, such as what are the institutional and organizational circumstances of the discursive event and how the nature of the discursive practice, and the constitutive/constructive effects of discourse are constructed in the social contexts.

**Figure 1:**
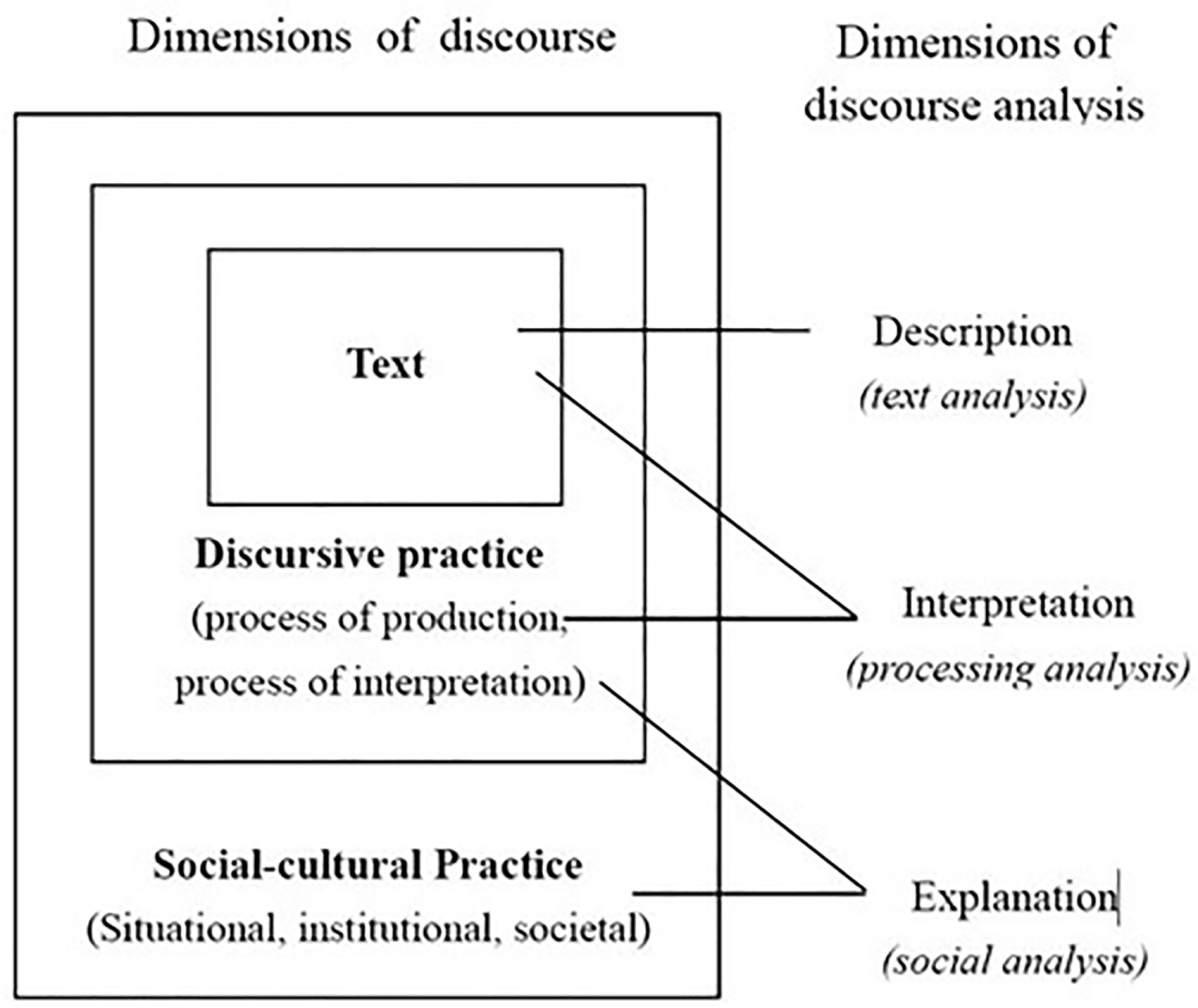
Fairclough’s three-dimensional model for discourse analysis.

As the linguistic representation of China’s diplomatic activities, Chinese diplomatic discourse has changed following China’s diplomacy over time, resulting in ever-changing political, economic, and social situations domestically and internationally. Accordingly, investigating Chinese diplomatic discourse in different periods can reveal the changes in China’s diplomacy over time. This research essentially explores the interaction between discourse and social changes, which fits Fairclough’s model well. Therefore, Fairclough’s model for discourse analysis is employed to analyze the interaction between Chinese diplomatic discourse and China’s diplomacy (Figure 1).

Using this model, this study analyzes Chinese diplomatic discourse from the three dimensions of text analysis, discourse practice, and social practice. First, in the dimension of text analysis, the essential keywords and keywords of Chinese diplomatic discourse are analyzed. The essential keywords are those “keywords with higher recurrence rates on the keywords list of a series of texts in the observation corpus” (Wei et al., 2005, p. 169). Thus, they are shared by all the texts in the corpus, reflecting the themes under constant focus. Therefore, examining the critical keywords of Chinese diplomatic discourse sheds light on the essential factors that China has been paying attention to in its diplomacy.

The keywords are lexical items whose frequencies are much higher or lower in the observation corpus than in the reference corpus (Scott and Tribble, 2006). The investigation of keywords is a critical approach to examining the language features and actual contents of texts; it is also a significant research area in corpus-based translation studies and corpus-based critical translation studies (Hu and Li, 2015). In this sense, examining the keywords of Chinese diplomatic discourse in different periods highlights the diplomatic priorities of China over time.

The dimension of discourse practice represents the discursive practice of China’s diplomatic strategies and foreign policies over time, such as the diplomatic subjects, objects, goals, scopes, and interests. Jin (2007) states that no diplomatic activity can be separated from the six elements of diplomacy: the diplomatic subject, object, foundation, purpose, method, and scope. Diplomatic strategies, the general plans for a country’s diplomatic activities, usually include diplomatic ideas, diplomatic interests, diplomatic strength, diplomatic objectives, and foreign policies. Different diplomatic entities naturally enact different strategies to meet their respective diplomatic objectives. As Jin (2007, p. 109) suggested, “for the same diplomatic entity, the specific contents of its diplomatic strategies are often adjusted and changed at the different historical periods.” As a discourse, the product of the social construction and discourse construction, influences society, we further analyze how Chinese diplomatic discourse reflects the social reality and changes in China’s diplomacy through the changes in diplomatic subjects, objects, objectives, scopes, and interests at the discourse level in different periods.

The dimension of social practice reflects China’s diplomatic strategies and foreign policies in different periods, which are determined by the social and historical conditions at that time. In other words, China’s diplomatic strategies and policies are aligned with political, economic, and social changes domestically and internationally. These constantly changing strategies and policies inevitably change China’s diplomatic objectives, purposes, modes, scopes, etc. Since these changes are represented linguistically through Chinese diplomatic discourse, it constantly evolves with changes in domestic and international environments. In this sense, the social, economic, and historical factors that prompt the changes in China’s diplomatic policies and strategies are explored, as these lead Chinese diplomatic discourse to change over time.

### Methodology and procedures

This study investigates the changes in China’s diplomacy by scrutinizing the Chinese diplomatic discourse and examine in the light of the rules of language use by analyzing regular linguistic patterns. Sinclair (1991) points out that using the corpus methodology to research a large number of texts systematically can reveal linguistic facts that may have thus far been neglected. In addition, the corpus approach is a more effective research method than other methods, and its results can be verified (Leech, 1993). As it is based on the linguistic data that people use, it is not subject to interference from the researcher’s personal intuition or judgment, ensuring that the research results are more realistic, reliable, and objective (Hu, 2011; Afzaal et al., 2022a,b). The procedure comprises three major steps. First, the key keywords of Chinese diplomatic discourse are examined. Seven keyword lists of Chinese diplomatic discourse and the keywords that appear five times or more are screened to create a major keyword list to examine the long-term focus of China’s diplomacy. Then, the keywords of Chinese diplomatic discourse are examined. Content keywords usually highlight the theme of the texts, while functional keywords reflect their style (Li and Hu, 2017). Therefore, the examination of the keywords of Chinese diplomatic discourse over time helps us identify the priorities that China values most in each period. Finally, in the dimension of social practice, the social and historical factors that contributed to the changes in China’s diplomacy are explored.

## Findings

This part illustrates the results of this research by examining the critical keywords of the Chinese discourse and the keyword of the Chinese discourse of seven different periods. As discussed above, essential keywords are those keywords that appear in all the texts in the corpus, which are the patterns repeatedly used in all texts to highlight the top priorities of China’s diplomacy all the time. Therefore, examining the critical keywords of Chinese diplomatic discourse in this section will first draw attention to the primary highlights of China’s diplomacy over the past 70 years. In addition, the keywords are lexical items with much higher or lower frequencies. Investigating the keywords of the Chinese diplomatic discourse of different periods will reveal the focus of China’s diplomacy at different periods. Accordingly, examining the keywords of the Chinese diplomatic discourse helps analyze the characteristics of China’s diplomacy at different periods and then further explore the Changes in China’s diplomacy over time. As a result, the scrutiny of the Chinese diplomatic discourse will, on the one hand, underline the long-term priorities of China’s diplomacy over the past 70 years and, on the other, highlight the primary mission of China’s diplomacy in different periods, respectively.

### Key keywords and China’s diplomatic stance

The keyword lists of Chinese diplomatic discourse are created using the British National Corpus as the reference corpus; the *p* value is set at less than 0.0001 and the cutoff point is set at 100. Then, the keywords that appear five or more times on these keyword lists are screened out and the top 20 are selected (Table 2).

**Table 2:** Key keywords of Chinese diplomatic discourse (1949–2018).

No.	Major keyword	Freq.	No.	Major keyword	Freq.
1	China	7	11	Taiwan	6
2	Chinese	7	12	We	6
3	Countries	7	13	Cooperation	5
4	People	7	14	Country	5
5	Relations	7	15	Friendship	5
6	World	7	16	Should	5
7	Asia	6	17	States	5
8	Our	6	18	United	5
9	Peace	6	19	US	5
10	Peaceful	6	20	Will	5

As shown in Table 2, 20 key keywords appear at least five times in the seven keyword lists and these can be tentatively categorized into four groups. The first group includes *China*, *Chinese*, and *we*; the second group includes *United States*, *US*, *Asia*, and *countries/country*; the third group includes *people*, *relations*, *world*, *peace*, *peaceful*, *Taiwan*, *our*, *friendship*, and *cooperation*; and the last group includes *should* and *will*.

The first group of key keywords (*China*, *Chinese*, and *we*) is the subject of China’s diplomacy, which implements its diplomatic policies and carries out its diplomatic activities and events. The concordance search results (19,608 concordance lines) show that these keywords are widely used in Chinese diplomatic discourse as the subject of China’s diplomacy. For example,

*“**We** are committed to building a harmonious world of lasting peace and common prosperity”*. (20070421)

The second group (*United States*, *US*, *Asia*, and *countries/country*) is the object of China’s diplomacy. Since 1949, the *United States/US* has been one of the most important objects for China’s diplomacy; meanwhile, China has attached great importance to diplomatic relations with countries in *Asia*, especially its neighbors such as Japan, South Korea, and India. For instance,

*“We actively supported the other developing **countries** in taking part in the Expo, including free participation for some **countries** from **Asia**, Africa and Latin America.”* (20101101)

*“China is the biggest trading partner of ASEAN, Japan, ROK, India and Australia and the biggest source of investment for many countries in the region.”* (20120705)

The third group (*people*, *relations*, *world*, *peace*, *peaceful*, *Taiwan*, *our*, *friendship*, and *cooperation*) is about the purposes and objectives of China’s diplomacy, namely, to establish good relations with other countries, safeguard world peace, build friendships with people from various countries, cooperate and promote mutual development, and properly handle the question of Taiwan. The Chinese people have suffered several wars (e.g., the War of Resistance against Japanese Aggression, the War of Liberation, World War II); therefore, a major task of China’s diplomacy is to safeguard world peace, while opposing war. This is repeatedly addressed on different occasions, such as,

*“In a globalized world where the interests and destinies of all countries are entwined, **peace** is what we all desire and **war** is what we all detest.”* (20100923)

China has been actively establishing good relations with countries worldwide. In 1949, it had diplomatic relations with about 10 countries, which had increased to over 170 countries by 2012 (Qi and Li, 2014).

The last category comprises the two modal verbs *should* and *will*, which reflect China’s inclination and attitude toward diplomatic affairs. Halliday (1994) classifies modal verbs into three types according to their value, namely, high, medium, and low. High-value modal verbs are used to represent positive and resolute attitudes, while low-value modal verbs are used for the opposite and medium-value ones are used between these two types. In this regard, *should* and *will* are medium-value modal verbs used in Chinese diplomatic discourse to express China’s positive and affirmative attitudes toward diplomatic affairs. For example,

*“We **should** abandon the zero-sum game and the Cold War mentality, make efforts to build a new type of international relations featuring win-win cooperation …”* (20160603)

In summary, the examination of the key keywords of Chinese diplomatic discourse reveals that the objects of Chinese diplomacy have always included the United States, the former Soviet Union, Japan, the United Kingdom, neighboring countries in Asia, and nations in Latin America and Africa. The goal of China’s diplomacy is to safeguard world peace and oppose war, establish good relations with people globally, and promote the common development of China and other countries. The scope of China’s diplomacy is expanding constantly. In addition, China has an active and affirmative attitude toward diplomatic affairs.

### Keywords and China’s diplomatic stance

To create the keyword list of each period, those keywords in the major keyword list (Table 2) are excluded and the top 20 (content) keywords of each period are selected (Table 3).

**Table 3:** Top 20 keywords in the seven periods.

First period (1949–1958)	Second period (1959–1968)	Third period (1969–1978)	Fourth period (1979–1988)	Fifth period (1989–1998)	Sixth period (1999–2008)	Seventh period (2009–2018)
Mao	Mao	Mao	Socialism	Hong	Development	Development
Soviet	Imperialism	Cambodia	Reform	Kong	Sides	International
Burma	Shek	Samdech	Hong	Development	International	Economic
Chiang	Chiang	Health	Kong	Economic	Mutual	Global
Shek	Kai	Indochinese	Socialist	World	Economic	Mutual
Imperialists	Japanese	Aggression	Mao	Sino-	Republic	security
War	Struggle	Nixon	Plenary	Reunification	EU	Sides
Kai	Japan	Sihanouk	Revolution	International	Exchanges	Exchanges
Union	Montgomery	Samdech Penn Nouth	Productive	Compatriots	Strengthen	ASEAN
Comrade	War	Norodom	Policies	Stability	Security	Promote
Stalin	Imperialists	Imperialism	Zedong	Motherland	Bilateral	Regional
Aggression	Vietnam	Cambodian	Mistakes	Straits	Promote	Africa
Korea	Troops	national	Policy	Developing	Stability	Growth
Kuomintang	Revolution	Modernizations	Principles	Socialism	Dialog	Efforts
Diplomatic	Snow	Japan	Modernization	Need	ASEM	Sustainable
Burmese	American	Zedong	Party	Defense	Regional	EU
Imperialist	Latin	Penn	Economy	HKSAR	Developing	Stability
Sukarno	Africa	Struggle	Years	APEC	SCO	Dialog
Zhou	Not	Normalization	Modernizations	Socialist	Efforts	Strengthen
Imperialism	Imperialist	Japanese	Shall	Rights	APEC	Bilateral

Table 3 shows that among the top 20 keywords in the first three periods of China’s diplomacy, many relate to international and regional politics, including the names of countries and regions such as *Soviet Union*, *Burma*, *Korea*, *Japan*, *Vietnam*, and *Cambodia* and the names of political figures such as *Mao Zedong*, *Chiang Kai Shek*, *(Joseph) Stalin*, *(Bung) Sukarno*, *Zhou (Enlai)*, *(Bernard) Montgomery*, *(Edgar) Snow*, *(Edward) Heath*, *Samdech Norodom Sihanouk*, *Samdech Penn Nouth*, and *(Richard) Nixon*. The keywords also include those highlighting the themes of war and struggle, namely, *imperialist (s)*, *imperialism*, *war*, *aggression*, *Kuomintang*, *struggle*, *troops*, and *revolution*. Hence, China’s diplomacy in the first three periods is characterized by its prioritization of politics over economy and others. This era is often referred to as the “politics-oriented” era (Liu, 2015).

Among the keywords in the first three periods, *Mao*, *Zedong*, and *Zhou (Enlai)* acted on behalf of China on the international stage as the subjects of its diplomacy, while the abovementioned countries and regions, as well as the political figures, are virtual objects of China’s diplomacy. These keywords suggest that at the founding of the People’s Republic of China, the country conducted most of its diplomatic activities with the two superpowers (i.e., the United States and the former Soviet Union), as well as some socialist countries and neighboring Asian countries.

In addition, the appearance of such keywords as *imperialist (s)*, *imperialism*, *war*, *aggression*, *Kuomintang*, *struggle*, *troops*, and *revolution* suggests that as a newly established country, China experienced wars and struggles both before and after its founding in 1949. To safeguard its sovereignty and national interests, it joined the socialist camp led by the Soviet Union and fought resolutely against such imperialists as the United States and Japan. Meanwhile, it, together with oppressed nations, launched revolutions and struggles against imperialist countries on the one hand and fought hard against Chiang Kai-shek and Kuomintang on the other.

Except for the abovementioned keywords, the remaining keywords in the first period (1949–1958) include *comrade*, *diplomatic*, and *Burmese*. Comrade, as a salutation originating from the Soviet Union, refers to people with common aspirations and it is often used to address strangers. However, it was used to distinguish between the two camps (socialists and imperialists) at that time. The concordance lines of *diplomatic* show that 71 of the 91 are found in the phrase “the establishment of diplomatic relations.” This illustrates that at the beginning of the People’s Republic of China, it attached great importance to the establishment of diplomatic relations with other countries. *Burmese* appears as a keyword because of the Sino-Burmese border issue. On 1 October 1960, Chinese and Burmese leaders signed the Boundary Treaty between the People’s Republic of China and the Union of Burma in Beijing, the first border treaty between China and its neighboring countries.

The remaining keywords in the second period (1959–1968) include *Japanese*, *not Americans*, and *Latin*. Among them, *Latin* mainly appears in the lexical items of “Latin America” and “Latin American countries.” At the beginning of the People’s Republic of China, efforts were made to establish diplomatic relations with other countries. Latin American countries, especially developing ones, were important objects of China’s diplomacy. Surprisingly, *not* is among the keywords used in this period. The concordance search results show that it is mainly used to express China’s stance and attitude toward foreign affairs, for example,

*“They do **not** agree with us, and we do **not** agree with them.”* (19590510)

The study uses AntConc version 3.5.7 to identify the collocates function. It is found that the keyword *Japanese* mainly appears in such expressions as “Japanese people,” “Japanese aggression,” “the Japanese warlord,” “Japanese friends,” “Japanese monopoly capitalists,” “the Japanese government,” “the Japanese imperialist,” and “Japanese militarism.” These expressions show that diplomatic relations between China and Japan were complicated at that time, including positive and negative aspects.

The remaining keywords in the third period (1969–1978) include *Indochinese*, *Cambodian*, *national*, *modernizations*, and *normalization*. The concordance search results show that most occurrences of *Indochinese* are in the expression of “Indochinese People against US aggression,” which highlights China’s advocacy that Indochinese people should unite to fight foreign aggression. In 1978, China held the third plenary session of the 11th Central Committee of the Communist Party of China, making the strategic decisions to implement the reform and opening up policy and shift its focus from class struggle to socialist modernization as well as the normalization of relations with Japan and the United States. This is why these three keywords (*national*, *modernizations*, and *normalization*) appear as keywords. Such statements are frequently seen in Chinese diplomatic discourse in this period, for example,

*“At present the Chinese and American people strongly desire the **normalization** of relations between the two countries and the relaxation of tension.”* (19720221)

Table 3 presents the keywords in the fourth and fifth periods are significantly different from those in the first three periods. The number of economy-related keywords is rising, while that of politics-related keywords has declined. In addition, with changes domestically and internationally, the theme of the time has changed from “war and revolution” to “peace and development.” As pointed out by Deng (1993), there were two global strategic issues at that time: peace and development. Among these two issues, development was the more important. The priority of China’s diplomacy during these periods aimed to promote national economic development, and it is often referred to as the “economy-oriented” era (Chen, 2012; Liu, 2015). In addition, the unification of Hong Kong and Macao, which was important for China’s diplomacy, meant that such keywords as *Hong Kong*, *reunification*, *motherland*, *compatriots*, *socialist*, *socialism*, and *HKSAR* appeared among the top 20 keywords, which suggests that China prioritized this in its diplomacy during these periods.

In the fourth period, there are still some political keywords such as *socialism*, *socialist*, *revolution*, *Mao Zedong*, *and Party*. Economy-related keywords such as *reform*, *productive*, *policies*, *policy*, *modernization (s)*, and *economy* were added into the list of the top 20 keywords. As mentioned above, China was shifting its priority from the establishment of the country to economic development and modernization. The concordance search result of the keyword *Plenary* shows that all its occurrences are in the lexical chain of “the third plenary meeting of the Central Committee of the Communist Party of China,” which marked the beginning of China’s reform and opening up period. This meeting has been highlighted as “a great turning point with far-reaching significance” (Yang, 2011, p. 43). In addition, the modal verb *shall* ranks 20th on the keyword list, which is used to indicate China’s positive and assertive attitude toward what is being discussed, for example,

*“We **shall** station troops there to safeguard our national security, not to interfere in Hong Kong's internal affairs.”* (19840622)

Among the keywords in the fifth period, in addition to economy-related keywords such as *development*, *economic*, *world*, *international*, *developing*, and *APEC* and those related to the reunification of Hong Kong and Macao such as *Hong Kong*, *reunification*, *compatriots*, *stability*, *motherland*, *straits*, *HKSAR*, and *rights*, the remaining keywords include *Sino*, *need*, and *defense*. The concordance search results show that all the occurrences of *Sino-appear* in the expressions of “Sino-US” (35 times), Sino-Japanese (14), Sino-Russian (9), Sino-African (9), Sino-Indian (4), Sino-Britain (2), Sino-Armenian (2), and Sino-Indonesian (1) to describe China’s relationships with these countries. The top five significant collocates (5: 5) of the keyword *defense* are *technology*, *national*, *active*, *strategy*, and *development*, which reflects China’s determination to strengthen its defense technology and capacity, for instance,

*“However, this in no way indicates that we can ignore technology. We need to work hard to develop **defense technology**; this is also an important strategic issue.”* (19910608)

Still, there is a modal verb *need* on the keyword list in the fifth period. The concordance search results show that it is used to indicate China’s attitude toward and need for economic development, such as,

*“… we **need** to keep economic development as our central task while promoting all-around social progress.”* (19980721)

As shown in Table 3, the top 20 keywords in the sixth and seventh periods are no longer limited to one or two specific aspects of China’s diplomacy such as politics and the economy, but are diverse, covering different aspects, including the economy, politics, security, cooperation and development, and global issues. Among the top 20 keywords in both periods, 16 are the same, namely, *development*, *international*, *economic*, *mutual*, *security*, *sides*, *exchanges*, *ASEAN*, *promote*, *regional*, *efforts*, *EU*, *stability*, *dialog*, *strengthen*, and *bilateral*. By category, politics-related keywords include *ASEAN*, *bilateral*, *sides*, and *regional*; economy-related keywords include *development*, *international*, *economic*, *mutual*, and *EU*; security-related keywords include *security*, *stability*, *dialog*, and *exchanges*; and development-related keywords include *strengthen*, *promote*, *cooperation*, and *development*. Some keywords appear in multiple categories, such as *development*, *strengthen*, *promote*, *EU*, and *efforts*. In this regard, China’s diplomacy is referred to as the new era of diplomacy (Jin, 2007; Liu, 2015).

As shown in Table 3, economy-related keywords such as *development*, *international*, and *economic* rank high on both lists, reflecting that economic development remains one of the top priorities in China’s diplomacy. Meanwhile, the frequent occurrences of keywords such as *EU*, *ASEAN*, *SCO*, *APEC*, *regional*, *bilateral*, *exchanges*, and *sides* indicate that China works with different international organizations and countries across regions to maintain international and regional cooperation and exchanges. In the 21st century, China actively promotes bilateral and multilateral diplomacy and contributes its wisdom and solutions to global governance through a series of diplomatic strategies, policies, and initiatives such as APEC, the Belt and Road Initiative, the Asian Infrastructure Investment Bank, and the so-called Major Country Diplomacy with Chinese Characteristics program. In addition, *security* and *stability* rank high on both keyword lists, which reveals that China attaches great importance to global and regional security issues as well as to maintaining *stability* in international and domestic situations to create favorable conditions for its domestic development, for example,

*“The heads of state supported building ties between the SCO and the Collective **Security** Treaty Organization and adopting coordinated efforts between the two organizations in maintaining **regional and international security** and addressing new threats and challenges.”* (20070823)

In addition, two verbs, *promote* and *strengthen*, appear as keywords on the list. To see their specific contexts, Table 4 lists their significant (mutual information value>3) collocations (5:5).

**Table 4:** Top 10 significant collocations of *promote* and *strengthen.*

Ranking	Collocation	Mutual information value	Frequency
1	And	5.17918	1,673
2	To	5.51722	1,022
3	The	4.04530	892
4	Cooperation	6.31602	602
5	Of	3.94285	457
6	In	4.49260	403
7	Development	5.44652	293
8	We	4.98734	202
9	Will	5.32409	169
10	Economic	5.44934	169

Table 4 shows that except for such functional words as *and*, *to*, *the*, *of*, and *in*, the significant collocations of *promote* and *strengthen* are *cooperation*, *development*, *we*, *will*, and *economic*. The frequent occurrences of these significant collocations indicate that China still emphasizes promoting and strengthening its economic development, which is crucial for its diplomacy.

The modal verb *will* shows the positive attitude of China and its frequent collocation with *promote* and *strengthen* forms a positive semantic prosody, which directly expresses China’s positive attitude toward and determination to promote and strengthen international and regional cooperation to ensure economic development through diplomacy. Moreover, the word *mutual* ranks fourth and fifth on the keyword list in these two periods. The search result for clusters 2–5 shows that it mainly appears in these four phrases: *mutual benefit* (230 occurrences), *mutual trust* (155), *mutual understanding* (114), and *mutual respect* (110). The high frequency of such expressions suggests that when dealing with international affairs, China attaches much importance to the benefits of its partners and values the trust, understanding, and respect between them, which is repeatedly addressed on different occasions, for example,

*“After fifty years of development, China-Arab relations have entered a period of maturity and stability and can boast a wealth of experience as follows: politically, **mutual respect** and equality; economically, **mutual benefit** and win-win cooperation; and culturally, **mutual** enrichment and complementarity.”* (20060531)

Except for the shared keywords, another four words are on the keyword list in the sixth period, namely, *Republic*, *developing*, *SCO*, *and APEC*. Among these, *Republic* mainly appears in the names of countries such as *the People’s Republic of China*, *the Republic of Tajikistan*, *the Republic of Korea*, *the Republic of Macedonia*, *the Republic of South Africa*, *the Federal Republic of Yugoslavia*, and *the Republic of India*, indicating that China has diplomatic exchanges with various countries. The concordance search results reveal that the keyword *developing* appears only in the two phrases of *developing countries* and *developing country*, which suggests that China, as a large developing country, values its cooperation with other developing countries and cares about their development, for example,

*“High priority should be given to boosting cooperation in human resources development and capacity building in the financial sector, especially financial capacity building for **developing countries**. **China** supports exploring the possibility of setting up a new financing mechanism for cooperation on the basis of the Asia-Europe Trust Fund to provide funding for future financial dialogue and capacity building.”* (20060910)

Another two keywords, *SCO* and *APEC*, are the names of international organizations in which China plays an important role. As repeatedly addressed by the members of the SCO and economies of APEC, the purposes and principles of these organizations are “to help consolidate regional security and stability and promote the common development of countries in the region” and “to reduce tariffs, improve customs efficiency, close the gap between developing and developed economies, combat climate change, dismantle terrorist networks, increase transparency, and stimulate economic integration.” As a member of international organizations, China participates in international affairs and contributes its vision and wisdom to global development, peace, security, and stability.

The remaining keywords in the seventh period (2009–2018) are *global*, *Africa*, *growth*, and *sustainable*. To see the context of *global*, clusters 2–5 are generated with AntConc 3.5.7 and the results show that the top 10 lexical clusters with the highest frequencies are *global economy* (244 occurrences), *global governance* (238), *global challenges* (222), *global economic governance* (218), *global development* (171), *global growth* (144), *global issues* (117), *global trade* (99), *global economic growth* (89), and *global financial crisis* (89). The frequent appearance of these lexical items in Chinese diplomatic discourse suggests that China, through its diplomacy, pays much attention to these global issues, particularly the global economy, global development, and global governance. This further indicates that it sees the world as having a shared future and that its diplomacy aims to address the issues that concern all people globally.

*Africa*, which also appears as a keyword on the list, has been one of China’s important objects since its founding in 1949. To see its context, its collocation list is generated with the Collocates function of AntConc 3.5.7 and the top five significant collocations are *China*, *cooperation*, *development*, *will*, and *relations*. These high-frequency collocations show that China values cooperation and relations with countries in Africa and strives to promote the development of the continent, for example,

*“**China** has done its best to help **Africa’**s development. Yet China is always grateful to **African countries** and people for their firm support and selfless help over the years.”* (20130325)

Further, *sustainable* and *growth* appear on the list of keywords for the first time. The concordance search results show that all the occurrences of *sustainable* are paired with *development* in the expression of *sustainable development*, describing China’s goodwill in its diplomatic relations, exchanges, and cooperation with countries globally, for example,

*“We are committed to green and **sustainable growth**, and addressing environmental challenges through closer practical cooperation.”* (20141112)

In summary, the investigation of the keywords in these seven periods shows that China’s diplomacy has evolved from being politics-oriented in the early days of its founding in 1949 to economic development being the main task since the reform and opening up period in 1978, and then to global sustainable development in the new era. Likewise, the scope of Chinese diplomacy has expanded from establishing diplomatic relationships with the former Soviet Union and other socialist countries, neighboring countries, and Latin American developing countries to maintaining diplomatic exchanges and cooperation with countries globally. The purpose of China’s diplomacy has also changed dramatically over time, as it aimed to achieve sovereign independence and restore its legitimate seat at the United Nations in the first three periods, to facilitate economic development and safeguard the peaceful reunification of Hong Kong and Macao with the Mainland in the fourth and fifth periods, and to actively participate in global governance to maintain global cooperation and development in politics, the economy, and security as well as the common progress and development of humankind in the new era.

## Discussion

The study unveils that language is a form of social representation that “can fully represent the status quo, development and changes in the society” (Di, 2011, p. 69). Chinese diplomatic discourse is the linguistic representation of China’s diplomacy and reflects its focus, which varies in accordance with the changes in the political, economic, and social conditions domestically and internationally. To achieve diplomatic purposes in different periods, China has adopted diverse strategies and policies to safeguard its national sovereignty and territorial integrity, create international conditions for national development, maintain world peace, and promote human progress.

The fundamental purposes of China’s diplomacy stated in the provisions of China’s constitutions and other documents and by the Chinese leaders on such international occasions as the General Assembly of the United Nations, are to safeguard national sovereignty and territorial integrity, to create international conditions for national development, and to maintain world peace and promote the progress of humankind (Qu and Zhong, 2012, pp. 1–2). The key keywords, as examined by this research above, together with their respective linguistic contexts, show that China has attached great importance to world peace and its sovereignty and territorial integrity, its economic development and the progress of the humankind.

The study results reveal that the top priority of China’s diplomacy is to safeguard China’s sovereignty and territorial integrity. Since its founding in 1949, China has taken the maintenance of national sovereignty and territorial integrity as the primary task of its diplomacy, and it has resolutely participated in several wars in the 1950s and 1960s to protect the sovereignty of China’s borders and the peace of the border areas. Furthermore, since the reform and opening in 1978, China has firmly established mutual respect for sovereignty and territorial integrity as the basic principle of bilateral relations while developing relations with western developed countries (Tian, 2020). China’s diplomacy encouraged other countries to recognize its independent and sovereign status and thus a legitimate diplomatic subject because “only countries with independent sovereignty can become a diplomatic subject” (Jin, 2007, p. 8). In 1949, China only forged diplomatic relations with the former Soviet Union and some socialist countries and adopted such diplomatic strategies as “Leaning to One Side” to seek their support. It also engaged in several wars, to provide a stable environment for domestic development. As the linguistic representation of China’s diplomacy, the keywords of Chinese diplomatic discourse of the first three periods, are characterized by the names of countries, leaders of states, political figures, and wars and struggles against imperialist countries and Kuomintang at the domestic and international levels in the first three periods. Accordingly, China’s diplomacy with this period (1949–1978) is characterized by war and struggle, and it is labeled as the “Politics-Oriented” period.

Another important mission of China’s diplomacy is to create international environment for China’s development and rejuvenation. As a nation just rose from the semi-colonial and semi-feudal society, China’s national strength was weak, and it had no other choice but to seek for international status, equality and cooperation through struggle when dealing with these world powers during 1950s to 1970s. After fighting with the western world for more than 20 years, China has successively won recognition of major western countries and established various constructive partnerships with them. Meanwhile, China’s cooperation with the Soviet Union also experienced twists and turns, and finally established a strategic partnership of equal cooperation on a new basis. In addition, China also handled its relations with developing countries in an equal manner, which helped China win their praises and opportunities for economic cooperation (Tian, 2020).

The maintenance of sovereignty and territorial integrity is only the basic premises for China’s economic and social development and rejuvenation, while the economic and social development requires the establishment of friendly and cooperative relations with various countries. It is suggested by Marx and Engels (1995, p. 695) that during social development, the economic factor is “fundamentally the decisive factor in the historical process.” as pointed out by Deng (1993) development is even more important than other problems and China adjusted its diplomatic policies and strategies to promote national economic development since the fourth period. Accordingly, it has adopted such diplomatic strategies as “non-alignment (*Bujiemeng*),” “keeping a low profile to make a difference (*Taoguang Yanghui*, *Yousuo Zuowei*),” and “insisting on keeping a low profile to actively make a difference (*Jianchi Taohui*, *Jiji Zuowei*)” to create an environment and opportunities for economic development. Since 1978, China has implemented its reform and opening up policy.

Through economic exchanges and cooperation as well as technological advancement, the Chinese economy has developed rapidly. The scope of China’s diplomacy has since expanded further, and China is now recognized as an independent sovereignty by most countries worldwide. Therefore, economy-related keywords have largely replaced politics-related ones to highlight the characteristics of China’s diplomacy, especially given the prioritization of economic development since 1978. Indeed, China has joined more than 130 intergovernmental organizations and signed over 300 multilateral treaties since then (Qu and Zhong, 2012, pp. 349–350), and the breadth and depth of its diplomacy has advanced. China’s economy experienced rapid growth within this period and China has become the 2nd largest economy in the world since 2011, and China’s diplomacy of this period (1979–2008) is characterized by its economic development, and this period in China’s diplomacy is labeled as the “Economy-oriented” Period.

China has long taken world peace and the progress of humankind as another vital mission of its diplomacy. China has been a victim of war of aggression many times in its history and has a strong desire for world peace. It has learnt from history that only in a peaceful international and domestic environment can it be free from wars between major countries, can it quickly heal the wounds caused by these wars, and can it win the historical opportunities for developing its economy and improving its people’s lives (Tian, 2020). Therefore, China has proposed many internationally recognized diplomatic principles, such as the Five Principles of Peaceful Coexistence, “to seek common ground while shelving differences” and “unity without uniformity” (Zhang, 2015, p. 197). Moreover, China has solved international disputes by applying these diplomatic concepts and principles and has achieved much with its foreign affairs policy. For instance, it has actively participated in UN peacekeeping operations to safeguard international peace and security. By February 2019, China had sent nearly 40,000 peacekeepers and completed more than 300-armed escort patrols. In addition, China promotes the progress of humankind through its diplomacy. China has been advocating the equality of all countries, the peaceful coexistence of countries with different social systems, the reform of the old international political and economic order, the democratization of international relations, the promotion of multi-polarity, the settlement of disputes between countries through consultation, the handling of international affairs through multilateralism, the establishment of a harmonious world of multiple coexistence, mutual benefit, and win-win results (Tian, 2020). Besides, China has continuously helped other countries, Albania and Vietnam in particular, achieve development and progress through its diplomatic measures and assistance. China’s assistance to Albania reached nine billion yuan from 1954 to 1978, equivalent to more than CNY 4,000 *per capita* (Qu and Zhong, 2012, p. 187). China also shares its development achievements and experience through diplomacy to promote economic development and achieve common progress. For example, it vigorously promotes such diplomatic strategies as the Major Country Diplomacy with Chinese Characteristics program, with an aim to build a shared future; develop relations with neighboring countries based on friendship, good faith, mutual benefits, and inclusiveness; and assist developing countries in Asia, Africa, and Latin America. China’s overarching aim is to provide its programs and wisdom to enhance global governance and benefit people globally, which helps present it as a responsible country. China’s diplomacy during this period (2009–2018) no longer focuses on politics or the economy. Instead, it has shifted to global cooperation and development to promote the progress of humankind, which scholars label the “New Era.”

## Conclusion

The study explored through the diachronic corpus of Chinese diplomatic discourse from 1949–2018 the long-term changes in China’s diplomacy over these 70 years from a discursive perspective. The examination of Chinese diplomatic discourse shows that the significant objects of China’s diplomacy include the United States, the former Soviet Union, Japan, the United Kingdom, and countries in Asia and Africa. China’s diplomacy aims to safeguard world peace while opposing war, promote development through cooperation, and establish good relations with people globally. The study identified that China’s diplomacy has constantly expanded from diplomatic exchanges with socialist and neighboring countries in the first three periods to communication and exchanges with more countries in the subsequent periods and global diplomatic exchanges and cooperation in various fields in the 21st century. Moreover, the content of Chinese diplomacy varies across periods. In the first three decades, establishing the country was the primary task of China’s diplomacy. Since its reform and opening up in 1978, the focus of China’s diplomacy has shifted to economic development. China’s diplomacy in the 21st century is global. Its priority is no longer on one aspect of national development but rather on safeguarding global stability and security, enhancing bilateral and multilateral cooperation, and promoting sustainable development worldwide. Hence, in different historical periods, China’s diplomacy has varied in its content, scope, and purpose depending on the domestic and international social, economic, and historical conditions, which is linguistically represented through Chinese diplomatic discourse.

## Data availability statement

The original contributions presented in the study are included in the article/supplementary material, further inquiries can be directed to the corresponding author.

## Author contributions

All authors listed have made a substantial, direct, and intellectual contribution to the work and approved it for publication.

## Funding

This research is supported by the major research project of “Construction, Translation and Communication of the Discourse of China’s major country diplomacy with Chinese characteristics” (grant number: 17ZDA319) of the National Social Science Fund of China and the major research project of “Construction and Comparison of China’s Image through Official Discourse of China, USA, UK, France and Russia” (grant number: 21ZD038) of the Social Science Fund of Hubei Province, China.

## Conflict of interest

The authors declare that the research was conducted in the absence of any commercial or financial relationships that could be construed as a potential conflict of interest.

## Publisher’s note

All claims expressed in this article are solely those of the authors and do not necessarily represent those of their affiliated organizations, or those of the publisher, the editors and the reviewers. Any product that may be evaluated in this article, or claim that may be made by its manufacturer, is not guaranteed or endorsed by the publisher.
